# The spatial biology of HIV infection

**DOI:** 10.1371/journal.ppat.1012888

**Published:** 2025-01-24

**Authors:** Kevin Hu, Thomas R. O’Neil, Heeva Baharlou, Paul J. Austin, Jackson F. Karrasch, Lara Sarkawt, Yuchen Li, Kirstie M. Bertram, Anthony L. Cunningham, Ellis Patrick, Andrew N. Harman

**Affiliations:** 1 The Westmead Institute for Medical Research, Westmead, New South Wales, Australia; 2 School of Medical Sciences, Faculty of Medicine and Health, The University of Sydney, Sydney, New South Wales, Australia; 3 Brain and Mind Centre, School of Medical of Medical Sciences, The University of Sydney, Sydney, New South Wales, Australia; 4 School of Mathematics and Statistics, Faculty of Science, The University of Sydney, Sydney, New South Wales, Australia; Fred Hutchinson Cancer Center, UNITED STATES OF AMERICA

## Abstract

HIV infection implicates a spectrum of tissues in the human body starting with viral transmission in the anogenital tract and subsequently persisting in lymphoid tissues and brain. Though studies using isolated cells have contributed significantly towards our understanding of HIV infection, the tissue microenvironment is characterised by a complex interplay of a range of factors, all of which can influence the course of infection but are otherwise missed in ex vivo studies. To address this knowledge gap, it is necessary to investigate the dynamics of infection and the host immune response in situ using imaging-based approaches. Over the last decade, emerging imaging techniques have continually redefined the limits of detection, both in terms of the scope and the scale of the targets. In doing so, this has opened up new questions that can be answered by in situ studies. This review discusses the high-dimensional imaging modalities that are now available and their application towards understanding the spatial biology of HIV infection.

## Introduction

The natural progression of HIV infection is characterised by viral propagation in activated CD4^+^ T cells and latent persistence in resting CD4^+^ T cells. Over time, if untreated, there is a severe decline in CD4^+^ T cell levels that gives rise to a state of immunodeficiency [1]. Though the advent of antiretroviral therapy (ART) has helped significantly to curb this progression by reducing viral loads to clinically undetectable levels, it is not curative. HIV remains distributed in latently infected reservoirs, especially in resting CD4^+^ T cells and macrophages. Consistent studies have shown that rapid rebounds in viral load occur following disrupted periods during treatment, in turn necessitating lifelong therapy [2]. With 40 million people currently living with HIV and more than 1.3 million new infections each year, issues associated with access and adherence to ART are important problems and advances in reducing the size or eventually eliminating the reservoir are desperately needed. Furthermore, there is a growing recognition that tissue inflammation can undermine the efficacy of ART when administered for pre-exposure prophylaxis (PrEP) meaning there is an urgent need to design novel therapeutic approaches to block transmission [3–5]. To address this, an improved understanding of the events that underpin the different stages of infection from transmission to persistence is required, especially in a human setting as human beings are the only natural hosts for HIV.

### Significance of spatial biology towards understanding infections

As initial infection, spread and latency of many viral diseases occurs in tissue, spatial biology can provide unique insights by considering the native tissue context in which viruses interact with host cells and mediate disease. It allows for the assessment of a) relevant structural morphologies, such as epithelial barriers, b) cell localisation in compartments, such as epithelium, dermis/lamina propria, lymphoid follicles and sub-mucosa, c) interactions between pathogens and various cell populations, d) trafficking events in response to specific chemokines and cytokines, and e) how all of these relate to disease pathogenesis. For example, cells that are located proximal to the luminal surface may be more poised to encounter pathogens while lymphoid follicles may provide a viral sanctuary to evade therapeutic compounds and therefore inadvertently perpetuate the disease.

In the context of HIV, studies using cells derived from cell lines, peripheral blood, or dissociated tissues have informed much of our understanding of the dynamics of infection. Although HIV can be transmitted through infected blood and from mother to child, the overwhelming majority of transmission occurs via sexual intercourse in the genital and anorectal tracts, and lymphoid tissues harbour almost the entire viral burden in the body [6]. It is in turn essential to account for the role that the tissue microenvironment may have during the course of infection, aspects of which can only be elucidated using *in situ* imaging methods and not cell-suspension based techniques [7]. Traditionally, such studies of HIV infection have been largely qualitative in nature and limited in their scope due to several factors. Firstly, immunohistochemical and immunofluorescence-based imaging techniques have been the standard for early *in situ* studies. Fluorophore spectral spillover and the cross-reactivity of secondary antibodies however impose restrictive parameter limitations so that only a handful of markers and relatively few cell types can be detected in any one sample. However, such low-plex platforms have the advantage of being utilised with automated staining platforms. Secondly, autofluorescence in tissues is a common occurrence and often leads to false identification of marker expression. Thirdly, antibody-based detection methods for HIV have insufficient sensitivity for low levels of virions, making it challenging to investigate events related to initial transmission or established latency. Over the last decade however, new imaging techniques or modalities have been developed that circumvent these issues, providing opportunities to further our understanding of the dynamics of HIV infection.

### Approaches to detect HIV *in situ*

**Fig 1:**
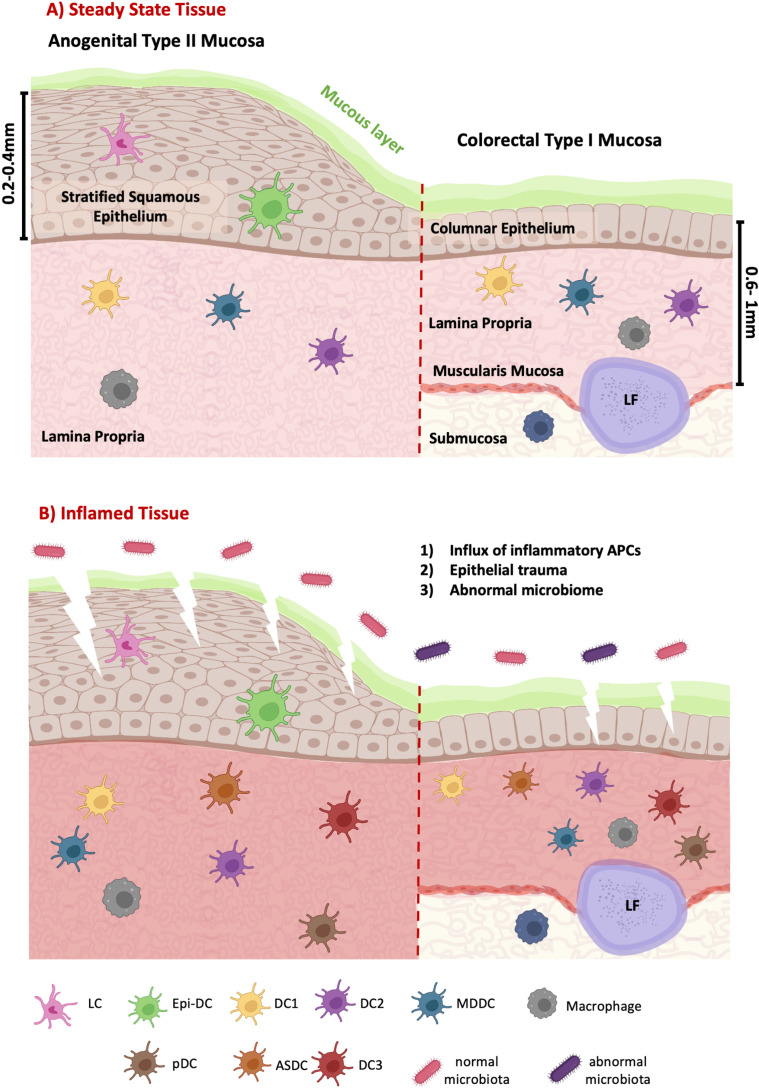
Schematic illustrating mucosal anogenital tissue compartments and the APCs they contain in both steady state and inflammation. The Type II epithelial layer of the vagina, ectocervix, inner foreskin, fossa naviculars and anal canal consists of a multilayered stratified squamous epithelium (SSE) of keratinocytes whereas the Type I mucosa of the colorectum consists of thin and fragile monolayer of columnar epithelial cells. Underlying both epithelial surfaces is a layer of connective tissue referred to as lamina propria. The colorectum contains thin muscle layer called the muscularis mucosa which separates the lamina propria from the deeper submucosa. This tissue also contains lymphoid follicles which can span the lamina propria and submucosa. A) Contained within the SEE of the Type II mucosa are both LCs and epi-DCs, but in contrast to the SSE of skin, DCs predominate. Contained within the lamina propria of both Type I and II mucosal tissues are DC1, DC2, MDDC and macrophages. Although the submucosa of the colorectum contains some DCs, macrophages are the predominant myeloid subset. B) Inflamed anogenital tissues are associated with increased HIV transmission for which there are three potential reasons: 1) these tissues contain additional inflammatory APCs including pDC and new discovered DC3 and ASDC which express higher levels of the HIV entry receptor CCR5; 2) inflamed tissues result in a more fragile epithelium which is more susceptible to mucosal abrasions allowing the virus to cross the epithelial barrier; 3) anogenital inflammation is often associated with an abnormal microbiome which secrete metabolites which break down PrEP drugs.

HIV is primarily transmitted via sexual intercourse and occurs through three key portals of entry. These are the cervix and vagina of the female genital tract, the foreskin and penile urethra of the male genital tract, and the anorectum. Across these tissues, the rectum is considered to have the highest probability of transmission per exposure event with a 15-fold and a 50-fold increase in likelihood compared to the vagina and foreskin respectively [8]. Contributing to this are the anatomical differences between tissues. The vagina consists of a type II mucosal surface that is comprised of non-keratinised stratified squamous epithelium which overlies a basement membrane and the lamina propria and its thickness varies with stages of the menstrual cycle. In foreskin, the epidermis consists of keratinised stratified squamous epithelium and is separated from the dermis by a basement membrane at the dermal-epidermal junction. By contrast, the rectum is lined with only a single layer of columnar epithelium above the underlying basement membrane, lamina propria, muscularis propria and submucosa [9]. This tissue is particularly unique in that it also consists of many dispersed lymphoid follicles (Fig 1), and many of the immune cells residing here express co-entry receptors of HIV [10,11].

Methods for imaging HIV/SIV *in situ* primarily rely on either whole-body or slide-based approaches. While this review focuses on slide-based imaging techniques, we briefly discuss whole-body imaging approaches here. Whole-body imaging of HIV/SIV distribution is achieved using ImmunoPET, a technique first described by Santangelo et al. In this method, metal isotopes with an ideal half-life (e.g., ^64^Cu, t₁/₂ = 12.7 hours) are conjugated to antibodies targeting the HIV/SIV envelope protein, ‘Env’ [12]. The radiolabelled antibodies are administered intravenously to infected animals, allowing adequate biodistribution before positron emission is detected using a PET scanner. Numerous studies have since utilized this approach, including two clinical trials in humans [13–18]. However, issues with reproducibility have emerged, with the two clinical studies producing conflicting results: one concluded that ImmunoPET could successfully detect HIV *in vivo* [18] while the other reached the opposite conclusion [17]. A recent preprint by Srinivasula et al. sought to reproduce and reconcile findings from these earlier studies [19]. Their work demonstrated that anti-Env probes exhibit significant non-specific binding both *in vivo* and in *ex vivo* tissue slices, ultimately questioning the validity of prior studies and the current utility of ImmunoPET as a tool for *in vivo* lentivirus detection.

**Fig 2:**
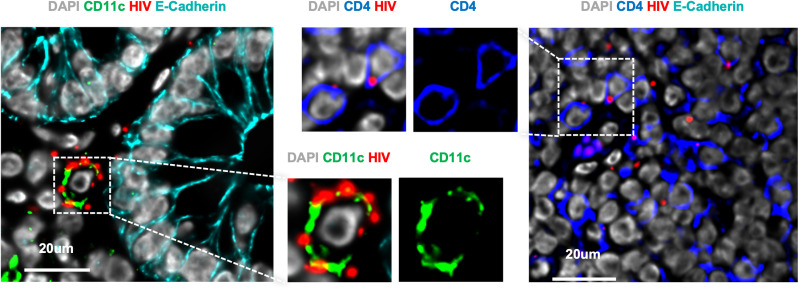
RNAScope detection of HIV_Bal_ (red) interacting with CD11c^+^ dendritic cells (green) and CD4^+^ T cells (blue). Images sourced from original publication [24].

At present, there are several different approaches that can be employed for the detection of HIV *in situ*. The first of these methods involves using antibodies to target proteins expressed by the virus such as the p24 protein that is expressed on the capsid surface. This approach typically has high levels of background staining, and its low sensitivity also means that it is reliant on the presence of virus signals which exceed specific thresholds following *de novo* replication. Alternatively, fluorescently labelled HIV such as GFP-Vpr labelled virus have been used in explant studies, though these can be difficult to distinguish from autofluorescence punctate signals. A variation of this is the use of photoactivatable HIV such as PA-GFP labelled virus where images before and after photoactivation with specific wavelengths of light are compared so that false positive signals can be subtracted [20]. It has been reported that this method sees up to a ten-fold increase in signal above background levels following photoactivation. A more widely adopted method is that of RNA *in situ* hybridisation (RNA-ISH), of which there have been several versions over the years. The current standard known as RNAscope significantly improves on previous generations of RNA-ISH regarding specificity, sensitivity, and the time needed to acquire images [21]. These can be attributed to its requirement for two target probes that bind to a contiguous RNA sequence before preamplifier molecules can bind to them. A branched chain signal amplification cascade is subsequently developed that allows HIV to be detected at single virion sensitivity with robust background suppression through chromogenic or fluorescent means (Fig 2). An advantage of this technique is the ability to spatially co-detect multiple different pathogens, as HIV is associated with a higher incidence of co-infections. By targeting the sense strand, this approach has further been shown to be able to detect single copies of viral DNA in the nuclei of infected cells and this modified technique is known as DNAscope [22,23].

### The use of imaging for *in situ* studies of HIV infection

#### Proteomic imaging.

Understanding and defining the structural differences between tissues provides an important basis for determining how the course of infection occurs in each tissue, which is one of the biggest advantages to microscopy. The various approaches to detect HIV *in situ* can be combined with imaging modalities to resolve the tissue landscapes during infection to different extents. As multiplexed proteomic imaging modalities become increasingly utilised, here we review previous *in situ* studies on HIV infection and discuss the shift from conventional imaging and qualitative image analysis to multiplexed imaging and more quantitative analysis in recent years. We explore how this has changed our understanding regarding aspects of HIV infection, and how this will continue to fill knowledge gaps that still exist.

#### Electron microscopy.

Electron microscopy was historically used to identify HIV as the causative agent underpinning AIDS [25,26]. Its capacity to investigate cells at ultrastructural resolution has allowed us to understand its viral structures and aspects of its lifecycle such as cell entry and cell-to-cell transmission amongst purified cells co-cultured with the virus [27,28]. In the context of *in situ* studies, electron microscopy has revealed that in foreskin explants, HIV infected cells form viral synapses with apical keratinocytes, leading to the budding of new viral particles. Additionally, Langerhans cells in the epidermis likely internalize the virus and directly transfer them to T cells across a synapse [29,30]. Electron microscopy has been particularly useful in validating morphologically the presence of virions in cells of other tissues such as vaginal Langerhans cells (now known to be dendritic cells (DC)) as well as urethral macrophages [31–37]. The use of electron microscopy extends to gut-associated lymphoid tissues where it has been used to similarly visualise infection and to classify virions as either immature or mature [38]. Interestingly, studies in these tissues have revealed that virological synapses are not an absolute requirement for cell-to-cell transmission between closely apposed infected cells as mature virions can accumulate in the space between them [38,39]. While electron microscopy can provide unique insights at the subcellular scale, it lacks the versatility to investigate tissues beyond what is morphologically distinguishable.

#### Immunohistochemical and immunofluorescent methods.

While conventional immunohistochemical and immunofluorescent methods have a lower resolution compared to electron microscopy, the ease of their use for investigating the expression of multiple markers has made them a common tool across many *in situ* studies. These methods have shed light on many processes occurring *in situ* which support the course of infection. For example, exposure of cervical explants to SIV inoculum can induce the expression of chemokines such as CCL3 and CXCL8 and these are associated with clustering of plasmacytoid DCs (pDC) and macrophages in the subepithelial layer. Downstream expression of similar chemokines by these cells creates a chemical gradient that is spatially correlated with the recruitment of CD4^+^ T cells [40]. At lower levels in the submucosa, mononuclear phagocytes have been observed and can take up virus using the lectin receptor CD169/Siglec-1. In relation to the vaginal tract, multiple studies have used immunohistochemical or immunofluorescent methods to show that mononuclear phagocytes can interact with fluorescently labelled virus early after inoculation [41,42]. Although many of these in the stratified squamous epithelium were originally thought to be Langerhans cells, recent studies have shown these have been misidentified and are in fact DCs [33–37,43]. These conventional methods have also helped to characterise some of the key targets for infection at this portal of entry which has been a long-standing goal. Many infected T cells have been found to co-express CD4, CCR6, and the transcription factor RORγt, suggesting the Th17 lineage is implicated in infection [44].

Studies in other tissues recapitulate the utility of these conventional microscopy techniques. For instance, CD4^+^ T cells, macrophages and DCs have been observed clustering together in proximity to the foreskin epidermis [45]. The significance of this is that these interactions are likely to favour viral transfer events between the cells and so perpetuate the disease at the site of transmission. Macrophages have been observed as the first cells to interact with HIV in the penile urethra and subsequently migrate from the epithelial compartment following viral exposure [46]. This likely explains why HIV transmission can still occur after circumcision which has otherwise been an open problem in the field. *In vivo* conventional DC2 (cDC2), monocyte-derived DCs (MDDC) and monocyte-derived macrophages (MDM) can also interact with HIV virions in the subepithelial layer within 2 hours post-inoculation [47]. In the colorectal tract, immunohistochemical and immunofluorescence methods have similarly helped to characterise some of the key targets of infection. For example, in the rectum and anal canal, HIV^+^ T cells phenotypically resemble Th17, while HIV^+^ DCs were phenotypically immature [48]. Another beneficial aspect of these types of *in situ* studies has been the ability to investigate the role of other factors that may be present during sexual transmission such as seminal plasma. We now know that seminal plasma can promote the accumulation of leukocytes at the border between the epithelium and lamina propria, increase the frequencies of intraepithelial DCs, as well as induce their migration to the apical surface meaning that there is an increased likelihood of viral encounters [49,50]. Intriguingly, this primarily occurs with R5-tropic HIV but not X4-tropic HIV [51].

These microscopy techniques have also been instrumental in identifying cellular reservoirs of the virus. For instance, in individuals on ART, CD206/MR-expressing macrophages in the urethra and CD69-expressing resident memory T cells in the cervix continue to harbour viral RNA and so need to be accounted for when trying to eliminate the reservoir [31,52].

Traditional immunostaining approaches have also been used to investigate interactions between LCs and peripheral nerves, and how neurons modulate HIV infection of LCs. LCs are known to have a direct role in regulating cutaneous innervation density [53], with the depletion of LCs reducing immunoreactivity of the pan-neuronal marker PGP9.5 and neuropeptide calcitonin gene-related peptide (CGRP). Likewise, denervation of the skin or peripheral nerve injury results in activation and increased expression of PGP9.5 by LCs [54–56]. CGRP is released from intraepidermal sensory nerves (i.e., nociceptors) in response to their activation by noxious stimuli or local inflammation and is critical for mediating neuroimmune interactions [57]. A series of *in vitro* and *ex vivo* studies by Ganor and colleagues has demonstrated CGRP acts on monocyte-derived LCs (CD1a^+^ Langerin^+^ E-cadherin^+^) to reduce HIV infection by multiple mechanisms, including increased Langerin expression and proteasomal degradation of HIV-1, and reduced cell adhesion and antigen presentation to CD4^+^ T cells [58–60]. It is therefore speculated that a reduction in CGRP levels due to neuropathy may aid HIV infection at later points and that CGRP may be important for limiting infection within the nervous system [58].

Immunohistochemical approaches have also been central to identifying HIV-1 reservoirs in the brain, and their contributions to neurological symptoms, such as dementia [61,62]. Although HIV does not infect neurons [61], laser dissection combined with labelling of HIV DNA, CD68 and GFAP, has confirmed macrophages/microglia and astrocytes are infected and contribute to neuropathology and dementia severity [62]. Moreover, HIV-infected and activated microglia are known to produce the neurotoxic kynurenine metabolite, quinolinic acid, further contributing to neuropathology and dementia [63]. The ability of HIV-1 to integrate into glial cells, as well as in macrophages and lymphocytes in the brain, suggests a permanent reservoir of provirus in the brain that creates a barrier for eradication and should be the focus of ongoing investigations with high-parameter approaches [64,65].

Despite the widespread use of non-neurotoxic ART, sensory neuropathy still affects around 40% of those with HIV [66,67]. Neuropathy has been investigated in skin biopsies from HIV patients by quantifying immunoreactivity of PGP9.5 to show a reduction in intraepidermal nerve fibre density [68,69]. Macrophages and T cells near cutaneous nerves in patients with HIV-neuropathy have been shown to increase expression of CX3CR1, and CCR2 & CCR5 respectively, suggesting a role for these cells in nerve damage [70]. Reduced cutaneous expression of CaMKK2, a protein important in neuronal repair, as well as increases in inflammatory purinergic receptors, P2X7R and P2X4R, have also been demonstrated to contribute to HIV neuropathy [71]. Activated astrocytes expressing GFAP^+^, and increased IL-1 *β* and TNF *α* levels, have been observed in the dorsal horn of the spinal cord of HIV-neuropathy patients with chronic pain, compared to those without pain, suggesting neuroinflammation is critical to pain in this cohort [72]. Based on these observations there is a strong rationale for high-parameter *in situ* studies using contemporary imaging techniques to study the roles of LCs, sensory nerve fibres and CGRP in HIV infection, and the pathogenesis of HIV neuropathy at peripheral and central nervous system sites

A general limitation of *in situ* microscopy studies is that results tend to be qualitative in nature. However, recently, a series of post-acquisition bioinformatic image processing algorithms have allowed quantitative image analysis to be performed on datasets acquired even with conventional modalities. For example, cell segmentation and subsequent quantification of CD4^+^ T cells across inner and outer foreskin samples to assess their relative susceptibility to infection has revealed greater densities in the inner foreskin [73]. Quantitative image analysis has also shown that epithelial DCs associate with more virions than Langerhans cells on average per cell [34]. In the lymph nodes, productively infected cells have been shown to be restricted within follicular structures in elite controllers while such cells in non-elite controllers are otherwise distributed across the paracortical region [74]. In elite controllers, CD8^+^ T cells also express greater levels of cytolytic effector molecules in proximity to infected cells [75].

Image analysis can reveal spatially based mechanisms that underpin infection and immune control *in situ*. Despite the insights that can be gleaned, the scope of these analyses is generally limited by the parameter restrictions associated with immunohistochemical and immunofluorescent methods, thus limiting the simultaneous measurement of a variety of cell types and functional markers.

#### Iterative approaches to fluorescence-based imaging.

Iterative approaches that build on existing fluorescence-based imaging techniques have also been developed to circumvent the restrictive parameter limitations. One of these methods is cyclic immunofluorescence or CyCIF which involves staining a tissue sample with a single round of antibodies that have been directly conjugated to fluorophores. Following image acquisition, the sample is photobleached to inactivate the fluorophores and in doing so, it allows for a new round of fluorophore-conjugated antibodies to be used for staining. Depending on the structural integrity of the tissue sample after each cycle, the continuation of this iterative process can greatly increase the number of markers that can be detected in it. Drawbacks of this method however are that autofluorescence can still be problematic for distinguishing true staining, and that image sets across cycles need to be computationally aligned before composites can be used for performing image analysis [76,77]. Variations of this method such as co-detection by indexing (CODEX) and ImmunoSABER instead use oligonucleotides as the conjugates for the antibodies so that the staining can be done all at once (up to 100 parameters). In the case of CODEX, fluorescently labelled complementary oligonucleotide strands are introduced in iterative rounds of detection and chemically stripped after imaging [78]. For ImmunoSABER, complementary oligonucleotide strands bind to the short sequence strand attached to the antibodies and are subsequently extended using repeated primer exchange reactions. These can act as binding sites for fluorescently labelled complementary strands, and so the greater the number of reactions, the greater the resulting signal amplification [79]. As with CyCIF, CODEX and ImmunoSABER are subject to similar limitations. The types of image analysis that can be performed with these techniques is similar to those described for mass cytometry-based imaging and can be applied to three-dimensional imaging using serial sections [80]. As such, they extend the potential insights that can be gleaned from tissue samples by microscopy.

The transmission of HIV in a human colorectal setting has historically remained unclear due to the difficulty in detecting the virus at such an early timepoint. A recent study by Baharlou et al addressed this by combining 7-plex CyCIF with RNAscope to investigate the interactions between HIV and broad immune cell populations in human colorectal tissue explants which were inoculated with clinically relevant transmitted founder HIV strains for two hours [24]. In contrast to past *in situ* studies on HIV transmission, computational image analysis was central to this study. The computational method, AFid was developed to remove contaminating autofluorescence [81], before segmenting and classifying the cells based on their marker expression, tissue location, and co-localisation with virus signal. Spatial analysis revealed that the virus was enriched amongst DCs in the mucosa and macrophages in the submucosa rather than CD4^+^ T cells. In support of this, increasing gradients of DCs towards virions were observed. Of all the compartments in the colonic tissue, lymphoid follicles contained the majority of virus, and this may be attributed to trafficking by DCs as an increasing gradient of DCs harbouring virus towards these sites was defined. Lymphoid follicles are well-established viral reservoirs, and it is tempting to speculate that this action by DCs may contribute to early seeding of the mucosal reservoir [2,82]. Through modelling of HIV density variation across compartments, lymphoid follicles were shown to act as conduits for HIV to bypass the muscularis mucosa and traverse into the submucosa where it is taken up preferentially by submucosal macrophages which have been shown to be functionally distinct to their mucosal counterparts [83]. By applying another computational method, SpicyR [84] to assess the effect of local HIV on cell community formation, HIV^+^ areas of colorectum were shown to contain clusters of HIV target cells (DCs, macrophages, and CD4^+^ T cells) but non-HIV target cells (CD8^+^ T cells) were not found within these clusters. This interesting observation suggests that specific clustering between cells may promote rapid viral transfer within the mucosa in the first minutes of transmission rather than later in lymph nodes. These insights into HIV transmission reiterate some of the capabilities that are exclusive to quantitative analysis of multiplexed imaging datasets, which make it a useful complement to other types of HIV studies. While this study was limited to the investigation of broad immune cell populations, another recent study has optimised CyCIF panels that can interrogate subsets of these groups [85], and so it can be determined whether specific changes during infection are directly attributable to any particular subset.

#### Mass cytometry-based approaches to multiplexed imaging.

The parameter limitations of standard imaging techniques are restricted to a handful of markers that neglects opportunities to gain complex insights regarding *in situ* events occurring during disease. The last decade or so has seen the development of several multiplexed proteomic imaging modalities that use different approaches to circumvent this issue, all resulting in detection of significantly more protein markers at single cell resolution than before. One such approach has been the use of metal-conjugated antibodies in mass-based imaging techniques such as imaging mass cytometry (IMC) and multiplexed ion beam imaging (MIBI). For both IMC and MIBI, tissue samples are stained with antibodies that have been directly conjugated with unique metal isotopes (up to 37 and 40 parameters, respectively), most of which are from the lanthanide series. Regions of interest are progressively ablated by either a laser beam as in the case of IMC, or a plasma ion beam as in MIBI which liberates the metal isotopes as secondary ions. The abundance of each ion is measured by a time-of-flight mass spectrometer and mapped back to the original positions in the tissue from where they were derived to generate images of the staining [86–88]. The narrow mass spectrums of each isotope minimise spillover across channels and so allows for the concurrent detection of up to forty different markers. Tissue autofluorescence does not occur and the isotopes display stability such that the stained samples can be stored long-term without any loss of signal intensity. While IMC has a resolution of 1μm, the resolution of MIBI can be as low as 300nm [89]. Caveats of these techniques are that tissue is lost after ablation and that isotopic impurities and metal oxides can introduce background signal which require filtering before performing image analysis [90].

As with other high-plex modalities, it is beneficial to perform quantitative *in situ* analysis on mass-based imaging datasets, given the large number of markers that can be detected across samples. To do this, single cells need to be segmented in the images using DNA-intercalator staining to identify cell nuclei. A pan cell membrane marker such as CD298 or Na^+^/K^+^-ATPase can be used to identify and segment adjacent and amorphous cells. Several machine learning-based approaches have become available for this task, including Cellpose [91], DeepCell [92], StarDist [93], and ilastik [94]. Each of these uses a different algorithm or model to computationally identify possible cells in an image and they display variable results depending on the image to be segmented. Post-segmentation, marker intensities can be attributed to each of the cells to generate a profile for classifying them into different phenotypic populations and to perform a range of analyses. Examples of this include profiling cell compositions in tissue regions or sample groups, charting pathogen distributions across the tissue, exploring cell-cell or host-pathogen interactions based on co-localisations, structural associations of cells or pathogens, changes in marker expressions, and investigating the formation of clusters of cells that may indicate or contribute to the disease. Three-dimensional imaging can also be performed using serial sections [95]. Evidently, mass cytometry-based imaging significantly expands the utility of microscopy compared to the previously discussed conventional imaging methods.

In the context of HIV, MIBI was recently used to spatially profile the tissue landscape of lymph nodes derived from SIV-infected and uninfected rhesus macaques [96]. Metal-conjugated oligonucleotide probes against viral DNA (vDNA) and RNA (vRNA) were used to distinguish between infected cells that were transcriptionally active (vDNA^+^ vRNA^+^) or silent (vDNA^+^ vRNA^-^) along with other markers to delineate structural features and cell identities were included in the staining panel. In turn, this allowed extensive mapping of diverse cell populations across the tissue. A key benefit of using multiplexed *in situ* approaches is that it allows a greater understanding of higher order coordination between cells during the disease. In this study, each cell was anchored to allow for the phenotypic assessment and clustering of the cells in its immediate vicinity and therefore establish cell neighbourhoods. In doing so, neighbourhoods co-enriched for macrophages, neutrophils, and CD8^+^ T cells were found to be predominant in SIV-positive regions as opposed to those co-enriched for macrophages and CD4^+^ T cells which were predominant in the SIV-negative regions. In support of the notion that the tissue is remodelled during infection, proximity analysis demonstrated that infected cells were typically close to B cells or macrophages. IL-10 was notably elevated amongst both these cell types, and this correlated with vRNA levels in the region of interest. Based on the co-occurrence of these markers, it is postulated that SIV promotes IL-10 production to create an immunosuppressive environment that is conductive to infection. Spatial analysis however also revealed that CD56 was elevated around infected cells that were transcriptionally silent, suggesting a role for natural killer (NK) cells in regulating viral transcription [96]. These types of analysis provide unique insights that can only be attained through *in situ* studies and are therefore necessary towards understanding HIV infection.

#### Spatial transcriptomics as a new frontier for imaging.

**Fig 3:**
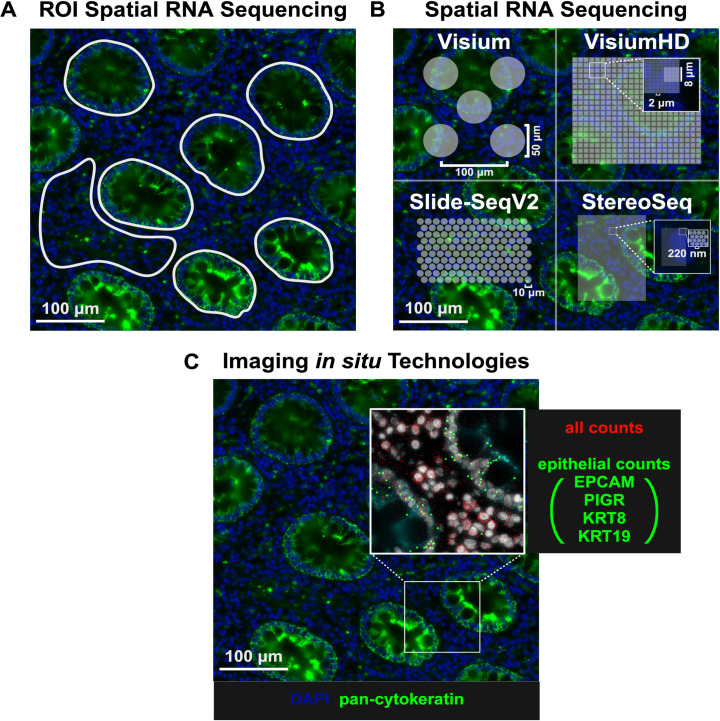
Representative fluorescent image of rectum mucosa stained with DAPI (blue) and pan cytokeratin (green). Illustrations of three spatial transcriptomic groups: A) Region of interest (ROI) spatial RNA sequencing (GeoMx), B) Spatial RNA sequencing (Visium, VisiumHD, Slide-SeqV2, StereoSeq), and C) imaging in situ hybridisation (CosMx, Xenium, Merscope). Created with Biorender.com.

Most recently, spatial transcriptomics has started to redefine the way in which we interrogate tissues, providing novel opportunities to explore *in situ* characteristics of diseases at the transcriptome level [97]. It can act both as a complement or as an alternative to proteomic imaging methods and can resolve tissues to different degrees of multiplexity and resolution depending on the commercial platform. In general, these can be classified into three broad groups (Fig 3) (Table 1) [98,99]. The **first** group of platforms can detect transcripts in regions with predefined sizes and it is the size of these regions that essentially determines the resolution. The GeoMx Digital Spatial Profiling System.

**Table 1:** Key features of spatial transcriptomic platforms.

Type	Example	RNA	Protein	Capture Size (Slide size)	Notes	
**ROI Spatial RNA sequencing**	GeoMx	Whole (NGS)	570+	10 – 600 µm user-selected ROIs (14.1 mm x 35.3 mm)	RNA of user-selected regions is collected and sequencing using NGS. Tissue may also be exposed to oligo-labelled antibodies or in situ hybridisation probes where UV light cleaves barcodes within the ROI	
					**Advantages**: Customisable panels Compatible with protein expression and TCR-sequencing High capture efficiency Comparatively cheap	**Disadvantages**: Fewer total ROIs Selection bias
**Spatial RNA Sequencing**	Visium	Whole (NGS)	Up to 36	55 µm spot (5 – 11 mm^2^)	Specialised slides contain barcoded probes dispersed across slides at varying sizes. Whole transcriptome sequencing is performed on mRNA captured in spots/tiles/beads. Unique probe sequences are then mapped to spatial coordinates. **Considerations:** Capture area, resolution, capture efficiency, multiomics capability, cost	
	VisiumHD		NA	2 µm tiles (8 µm bins) (6.5 mm^2^)		
	Slide-SeqV2		NA	10 µm beads (3 - 10 mm^2^)	**Advantages**: Spatially unbiased sequencing of large tissue regions Compatible with protein expression and TCR-sequencing Can reach single cell resolution	**Disadvantages**: Costly Tissue sacrificed during acquisition
	Stereo-Seq		100+	220 nm nanobeads (5 - 130 mm^2^)		
**Imaging** ***in situ* Technologies**	Xenium	Up to 5000	NA	NA (472 mm^2^)	Imaging spatial transcriptomic approaches utilise cyclic *in situ* hybridisations. Tissues undergo multiple rounds of probe hybridisation, fluorescent amplification, imaging and destaining.	
	CosMx	Up to 6000	Up to 68	NA (20 mm x 15 mm)	**Advantages**: Subcellular resolution across large areas Compatible with protein expression High specificity and sensitivity	**Disadvantages**: Costly and labour-intensive Smaller, predefined panels
	Merscope	Up to 1000	Up to 5	NA (Up to 9 cm^2^)		

ROI, region of interest; NGS, next generation sequencing; TCR, T cell receptor.

 (DSP) from NanoString for example uses probes with photocleavable oligonucleotide barcodes that bind to transcripts present in cells across the sample. As the dissociated oligonucleotides are measured according to manually selected region of interest, readouts from this platform indicate which transcripts were present in each of the regions of interest and their level of abundance [100]. The GeoMx DSP is limited to either micro-dissecting specific tissue compartments, sacrificing single-cell resolution, or selecting single cells, which results in a loss of spatial information. In the **second** group of commercial platforms, continuous arrays of oligonucleotides are distributed across the slide. The Visium platform from 10X Genomics performs transcriptome profiling using a grid array of capture areas 55µm in diameter which capture tissue mRNA with barcoded oligonucleotides that are subsequently used to generate cDNA for sequencing. This platform can be used to perform whole-transcriptome profiling across the entire tissue section. TCR sequencing is also compatible with some platforms, allowing for the study of T cell clonality and antigen specificity, particularly in determining the infected cells that undergo viral reactivation and the antigenic stimuli that triggers this process. However, Visium lacks single cell resolution, and the capture areas are roughly 100µm apart, leaving a 45µm gap of unused space. Alternatively, VisiumHD captures RNA with millions of barcoded tiles (2 µm), Stereo-seq chips with DNA nanoball-patterned arrays (220 nm), and Slide-SeqV2 with indexed beads (10 µm), significantly reducing the probe sizes such that the resolution approaches single cell level while still being able to interrogate the whole transcriptome [101,102]. However, these platforms have a lower capture efficiency, and tissue is sacrificed during acquisition, limiting the ability to further investigate the same tissue section, such as use of RNAscope to detect virus. Contrasting the first two groups, which leverage next generation sequencing via distributed probes of variable size, group **three** maps the precise subcellular positions of transcripts using *in situ* imaging to detect specific probes. This group includes the platforms such as Xenium from 10X Genomics, the CosMx Spatial Molecular Imager (SMI) from NanoString, and Merscope from Vizgen [103,104]. These platforms probe for mRNA from pre-designed panels of between 1000 - 6000 genes. However, custom probes are also becoming increasingly available such that panels can be tailored to the experimental requirements of users. It is thus feasible to detect intracellular and extracellular viral localisation, as has been proven with SIV probes using CosMx [105]. By co-imaging the samples with a nucleus stain such as DAPI and a pan-membrane marker such as CD298 or Na^+^/K^+^-ATPase, cells in the acquired images can be accurately segmented with machine learning models or algorithms. A key distinction of the third group is that downstream imaging can be performed. This is thus also compatible with the use of high-resolution fluorescent virus detection methods to identify the spatial and transcriptomic dynamics of cells that interact with HIV.

Recently, a variation of spatial transcriptomics that has been termed spatial metatranscriptomics has been reported, which allows for co-profiling of both the host transcriptome and microbiome [106]. This has important consequences as it further extends the scope of spatial transcriptomics and means that we will be able to also clarify the relationship between the tissue microenvironment and the microbiome, and the impact on the initial and progressing viral infection. Another emerging platform involves the combination of spatial transcriptomics with metabolomics [107]. The application of Spatial Multimodal Analysis could reveal the metabolic pathways that are active in productive and latent infected cells during *in situ* transmission, or during reservoir reactivation compared to those that remain latent.

A range of analyses can be performed on datasets derived from spatial transcriptomics, particularly those attained at single cell resolution, and these make it a powerful tool for investigating the dynamics of infection. For example, cells can be classified into distinct populations based on their transcriptional identities and mapped across the tissue to investigate preferential localisation across compartments in the absence and presence of disease. Alternatively, similar cell types in different tissue areas (such as virus-positive and virus-negative areas) can be compared for differential gene expression. This is particularly useful for gaining clues as to how a cell type contributes to or is affected by a disease, as well as to select biomarkers for the identification of infected cells. The interactive or migratory activities of cells can also be investigated from a number of angles such as by proximity analysis to other cells and structures, by ligand-receptor analysis, or by trajectory analysis similar to those routinely used in single cell RNA-sequencing studies [108], but with additional spatial modelling [109,110]. Cell networks can also be explored to identify coordinative processes which may mediate disease progression. Additionally, many of the available platforms provide protocols for immunofluorescent imaging and may be optimised to include viral detection by the aforementioned proteomic imaging methods.

### Considerations for HIV probe design

HIV/SIV detection, whether by RNAScope or as part of newer spatial transcriptomic platforms, requires the design of custom probes. Although HIV mutations occur at a high rate, one can design probes targeting relatively conserved regions such as gag and pol which is usually sufficient to detect single virions [22]. RNA degradation due to tissue fixation and staining procedures is another consideration. However, designing probes that span near the entire ~10,000 base pair HIV genome produces sensitive and specific signals [22]. More recent spatial transcriptomic platforms such as CosMx and Xenium are confined to a more limited range of a few hundred base pairs per unique channel making region selection for probe design a greater consideration. However, this can be circumvented by dedicating other unique channels to the detection of additional regions of the virus. This was demonstrated in an unpublished study in SIV infected macaques acquired using the CosMx platform [105]. Here, specific regions of SIV RNA such as gag, pol, nef, env, vif, and tat/rev/vpr/vp (as one amplicon) were probed using separate channels, thus increasing the sensitivity of SIV detection and allowing for finer analysis of individual SIV genes.

Identifying integrated HIV provirus is critical for HIV reservoir studies. As mentioned earlier, this has been achieved with the RNAScope platform, using probes targeting the sense strand with putative latent cells defined as HIV DNA^+^, HIV RNA^-^, and infected transcriptionally active cells defined as HIV DNA^+^, HIV RNA^+^ [22,23]. However, there are many challenges with this approach. First, there is only a single copy of integrated HIV provirus per cell. Second, provirus is inherently more difficult to access due to its nuclear location and strong interstrand forces in dsDNA which need to be broken to allow probe binding. Third, co-detection of HIV RNA and DNA requires that each probe targets distinct non-overlapping regions to prevent probe-probe dimerization, thus introducing a further constraint to an already challenging task of single molecule detection. Fourth, most integrated proviruses are defective, and this cannot be discerned with DNAScope, instead requiring full-length viral sequencing, currently only possible in single cells [111]. For these reasons, it is both technically challenging and unlikely that this approach can accurately measure the viral reservoir, particularly in ART suppressed individuals. However, it can be useful to study host-virus interactions during either initial infection or reactivation from latency, the former having already been studied in SIV infected macaques [96] as discussed earlier.

Detecting HIV/SIV RNA in platforms like Visium, which capture RNA via the common poly-A tail, presents challenges. Although HIV’s 3′ poly(A) sequence allows capture, the platform is not optimized for single copy or long RNA capture. Large RNA molecules, like the HIV genome, are prone to fragmentation during fixation and staining, limiting sequencing to regions near the 3′ poly(A) tail. This typically includes the 3′ Long Terminal Repeat, nef, and the highly mutation-prone Env gene [112]. These factors make HIV/SIV detection with 3′ poly(A) capture techniques challenging. Instead, these techniques can be used for spatial characterisation of key tissue compartments such as lymph nodes between infected and uninfected individuals, without probing for virus, as was performed in a recent unpublished study [113].

### How can high-dimensional imaging be used to address open problems in the HIV field?

Despite the advances in our understanding of HIV infection, significant progress is still required to develop novel therapeutics that will aid the eradication of HIV. To achieve this, there are several open questions that remain to be answered. Firstly, it is unclear how inflammation in the colorectal tract predisposes individuals to a greater risk of HIV acquisition [114,115]. The onset of inflammation at these sites may be due to a number of reasons, such as mucosal trauma, co-infection with other sexually transmitted diseases [116], or contact with seminal plasma [117]. It has been postulated that this may disrupt the epithelial barrier and thereby facilitate HIV access to subepithelial layers. Alternatively, inflammation could confer changes to the tissue composition of cells or even the way they respond to HIV. For example, proinflammatory cytokines can in some cases increase viral replication in Langerhans cells or promote their capture of viruses and subsequent trans-infection of CD4^+^ T cells [118]. Another critical problem relates to the comprehensive definition of the repertoire of immune cells that interact with HIV. Although it has been established that mononuclear phagocytes are amongst the first cells to encounter HIV at the portals of entry, the relative involvement of different mononuclear phagocytes is unclear, in part due to the diversity of populations but also the complexity attributed to ongoing changes in the definitions of their subsets. For example, Axl^+^ Siglec-6^+^ DCs (ASDC) were only recently identified but have nonetheless been found to be able to take up, replicate and/or transfer HIV [119–122]. Similarly, CD1c^+^ CD163^+^ DCs (DC3) have recently been defined in inflamed tissues but their interactions with HIV have yet to be investigated. In a similar sense, the repertoire of cells which are latently infected with HIV is yet to be entirely elucidated [123], and is a challenging task given that HIV can disseminate to many different tissues beyond the portals of entry and the circulatory system [6].

Sensory neuropathy is a debilitating complication of HIV infection. Utilizing high-dimensional imaging technologies will provide insight into the specific mechanisms implicated in its pathogenesis and pave the way for targeted therapeutics. Visualization and characterization of cutaneous nerve fibers lends itself to proteomic approaches like imaging mass cytometry or immunofluorescence, primarily due to their fine, punctate morphology and the fact that neuronal cell somata are located in the dorsal root ganglia (DRG). To date, spatial transcriptomic studies on the nervous system to date have focused on the DRG [124,125] or CNS tissues, such as the spinal cord or brain [126–128]. A combined approach of high-resolution spatial transcriptomics to assess immune cells and immunofluorescence for nerve fiber characterization in mucosal tissues or skin biopsies would be ideally suited to these investigations.

**Fig 4:**
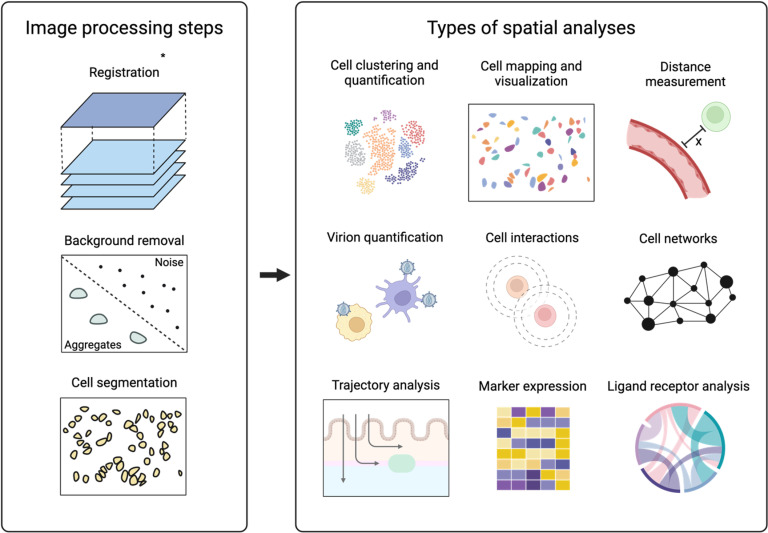
Image processing steps and types of spatial analyses associated with high dimensional imaging datasets. *Registration of images is a processing step that is specific to iterative fluorescence-based modalities such as CyCIF, CODEX and SABRE due to possible slide misalignments during image acquisition with every cycle. Created with Biorender.com.

As described previously, multiplexed proteomic imaging and spatial transcriptomics greatly expand the capabilities of *in situ* studies and the insights that can be drawn from them (Fig 4). These modalities are integral towards progressing the field of HIV research and can be used to address each of the open questions that have been mentioned. To illustrate this, we can consider the question regarding how inflammation predisposes individuals to a greater risk of HIV acquisition. Spatial transcriptomics and metatranscriptomics can be performed on vaginal tissue explants which have been pre-treated with herpes simplex virus and then topically infected with a clinically relevant strain of HIV to simulate a transmission event. Post-acquisition, the cells can be segmented and clustered to identify whether there are greater proportions of conventional target cells in the inflamed samples or if they express higher levels of HIV binding receptors. By quantifying the co-localised expression of HIV, we can also determine whether there are unconventional cell types that can harbour HIV under inflammatory conditions and therefore need to be accounted for therapeutically. Trajectory analysis based on changes in cell density can also provide information as to how the cells migrate and whether there is any preferential route that they take. Additionally, ligand-receptor and neighbourhood analysis can collectively shed light on common networks between cells which may promote infection but are otherwise difficult to delineate through qualitative means alone. Finally, differences in the microbiome can also be assessed and correlated with the rest of the spatial dataset. The presence of particular bacterial species such as *Prevotella sp* may elicit changes in the cytokine secretion profile of proximal cells and contribute to disease progression. Evidently, *in situ* studies such as these can provide a wealth of information regarding what is occurring at the tissue level and inform a more complete picture of HIV infection. Other examples of *in situ* studies to address open questions in HIV research are listed in Table 2. While we note the current paucity of these types of studies in the literature and the costs associated with them, we anticipate that there will be a greater adoption of high-dimensional imaging modalities over the following years. The establishment of an open and reliable biobank of rare human tissues would facilitate the diverse and necessary HIV research that address the remaining open questions.

**Table 2:** Examples of open questions in HIV research and *in situ* study designs to answer them.

Open Question	Study Design
What is the repertoire of immune cells that interact with HIV?	Use high resolution spatial transcriptomics on explants topically infected with clinically relevant strains of the virus. Identify interactions between immune cells and HIV based on co-localisations and determine the phenotypes of the cells using their transcriptional profiles. Repeat across samples derived from different portals of entry (e.g., cervix, vagina, foreskin, and colorectum). DNAscope and RNAscope can be used in multiplexed proteomic imaging to also identify and profile cells which are latently infected.
Why are some lymphoid follicles preferentially seeded with virus but not others?	Use three-dimensional spatial transcriptomics on explants topically infected with clinically relevant strains of the virus. Compare the phenotypes of cells in lymphoid follicles that have been seeded with virus to those without any virus. Explore cell neighbourhoods around the lymphoid follicles to identify any differences that may also explain this observation.
How do different inflammatory stimuli affect the course of transmission?	Treat tissue samples with different sources of inflammation (e.g., co-culture with herpes simplex virus, Prevotella sp or seminal plasma). Topically infect with clinically relevant strains of the virus at serial timepoints. Use high resolution spatial transcriptomics to infer a spatiotemporal model of the infection. Repeat across samples derived from different portals of entry (e.g., cervix, vagina, foreskin, and colorectum).
How do novel therapeutics against HIV affect the host?	Use high resolution spatial transcriptomics on explants treated with or without the novel therapeutics. Explore the phenotype, quantity, and distribution of immune and non-immune cells in the tissue. Investigate ligand-receptor profiles and higher-order cell networks to identify any dysregulations which may have been induced. Spatial metatranscriptomics can be used to determine if there are any profound changes amongst the microbiome.
How do intraepidermal nerve fibres modulate HIV infection in human explants?	Use high resolution spatial transcriptomics combined with nerve fibre immunofluorescence, and/or imaging mass cytometry (see Fig 5), on explants topically infected with clinically relevant strains of the virus to determine changes in neuropeptide expression (i.e., CGRP) in nerves and markers of LC function. Characterise effects of exogenous CGRP and inflammatory stimuli on nerve fibres and LC function in infected explants.
How do neuroimmune interactions contribute to sensory neuropathy in HIV?	Use high resolution spatial transcriptomics combined with nerve fibre immunofluorescence, and/or imaging mass cytometry, on skin biopsies from patients with and without HIV sensory neuropathy to investigate proximity of immune cells and nerve fibres. Investigate differentially expressed transcripts, especially chemokine receptors and immune cell activation markers that may contribute to nerve damage.

**Fig 5:**
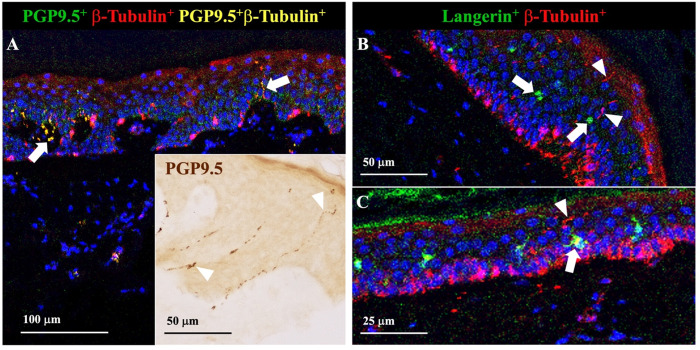
Imaging mass cytometry of intraepidermal nerve fibres. A) Representative image of cutaneous nerve fibres visualised using imaging mass cytometry (IMC). An indirect immunolabelling technique for PGP9.5 (green) and Class III β-Tubulin (red) combined with heavy metal-isotype conjugated tertiary antibodies was used to detect double-positive (PGP9.5^+^ β-Tubulin^+^, yellow) nerve fibres and to distinguish neuronal tissue from melanocytes, which also express Class III β-Tubulin, in the basal epidermis. An intraepidermal nerve fibre (white arrow, right) exhibiting a typical punctate morphology is seen extending from the stratum basale to the stratum granulosum. A thicker dermal nerve (white arrow, left) located within a dermal papilla is also visible. A, Inset) Representative image of intraepidermal nerve fibres (white arrowheads) detected by chromogenic immunohistochemistry for PGP9.5 (brown). B, C) Representative images of cutaneous nerve fibres and Langerhans cells visualised using IMC. Single-labelled (β-Tubulin^+^) intraepidermal nerve fibres (white arrowheads) are present in the stratum spinosum and stratum granulosum. Profiles of nerve fibres in the papillary dermis are also seen (not indicated). The Langerhans cells (white arrows) present in the epidermis are in close proximity to intraepidermal nerve fibres. For all IMC experiments, Zamboni’s-fixed, paraffin-embedded 3 mm skin punch biopsies were sectioned at 7μm and immunolabelled with heavy metal isotope-conjugated antibodies targeting proteins of interest (or tertiary antibodies in the case of indirect staining). Cell nuclei were labelled with Cell-ID Intercalator-Ir (Standard BioTools) and are indicated in blue in all three images. Acquisition was performed on a Hyperion+ Imaging System (Standard BioTools) and data was visualised in MCD Viewer (Standard BioTools). Abbreviations: IMC, Imaging Mass Cytometry; PGP9.5, Protein Gene Product 9.5; β-Tubulin, Class III β-Tubulin.

## Concluding Remarks

Despite the availability of ART, HIV continues to be a global health issue as the virus persists in individuals already living with the infection while new cases continue to be reported each year. Novel therapeutics are therefore needed to avert new transmission events and eliminate HIV. As the field of spatial biology rapidly advances, its potential for resolving the events that contribute towards the course of infection has concurrently expanded. Current imaging techniques are capable of profiling tissue microenvironments at both the proteomic and transcriptomic level. The application of these high-dimensional imaging modalities towards explant models of infection or biopsies derived from individuals living with HIV can in turn allow us to answer key questions relating to the dynamics of HIV infection *in situ*. Collectively, a greater understanding of the complex interplay between HIV and the immune response will be beneficial towards expediting the development of improved strategies to curb HIV transmission, infection, and persistence.
